# Control of *Foxo1* Gene Expression by Co-activator P300*

**DOI:** 10.1074/jbc.M113.540500

**Published:** 2013-12-30

**Authors:** Anne R. Wondisford, Lishou Xiong, Evan Chang, Shumei Meng, David J. Meyers, Mingsong Li, Philip A. Cole, Ling He

**Affiliations:** From the ‡ Division of Metabolism, Department of Pediatrics and; § Pharmacology and Molecular Sciences, Johns Hopkins University School of Medicine, Baltimore, Maryland 21287 and; the ¶ Digestive Division, Nanfang Hospital, Southern Medical University, Guangzhou 510515, China

**Keywords:** CREB, *Foxo*, Gluconeogenesis, Histone Acetylase, P300

## Abstract

FOXO1 is an important downstream mediator of the insulin signaling pathway. In the fed state, elevated insulin phosphorylates FOXO1 via AKT, leading to its nuclear exclusion and degradation. A reduction in nuclear FOXO1 levels then leads to suppression of hepatic glucose production. However, the mechanism leading to expression of *Foxo1* gene in the fasted state is less clear. We found that *Foxo1* mRNA and FOXO1 protein levels of *Foxo1* were increased significantly in the liver of mice after 16 h of fasting. Furthermore, dibutyrl cAMP stimulated the expression of *Foxo1* at both mRNA and protein level in hepatocytes. Because cAMP-PKA regulates hepatic glucose production through cAMP-response element-binding protein co-activators, we depleted these co-activators using adenoviral shRNAs. Interestingly, only depletion of co-activator P300 resulted in the decrease of *Foxo1* mRNA and FOXO1 protein levels. In addition, inhibition of histone acetyltransferase activity of P300 significantly decreased hepatic *Foxo1* mRNA and FOXO1 protein levels in fasted mice, as well as fasting blood glucose levels. By characterization of *Foxo1* gene promoter, P300 regulates the *Foxo*1 gene expression through the binding to tandem cAMP-response element sites in the proximal promoter region of *Foxo1* gene.

## Teaser

**Background:** The mechanism driving hepatic gene expression of *Foxo1* in the fasted state remains unclear.

**Results:** Activation of cAMP-PKA pathway induces *Foxo1* gene expression through CREB and co-activator P300.

**Conclusion:** P300 mediates *Foxo1* gene expression by binding to *Foxo1* proximal promoter.

**Significance:** Induction of the *Foxo1* gene by cAMP-PKA via P300 fully activates the gluconeogenic program during fasting to maintain euglycemia.

## Introduction

The maintenance of blood glucose levels within a defined range (70∼110 mg/dl, fasting) is critical in protecting organisms against hypoglycemia during fasting and hyperglycemia during feeding given that glucose is a major energy source for mammalian cells and is the sole energy source in some mammalian tissues and cells such as neurons and erythrocytes. Hypoglycemia induces damage to tissues and cells and can even cause cell death (1, 2). However, hyperglycemia also causes serious adverse effects to mammals through nonenzymatic glycosylation of many cellular proteins; these adverse effects are evident in patients with diabetes mellitus who suffer from microvascular damage to the kidney, retina, and nerves (3, 4). In response to elevated blood glucose levels in the fed state, pancreatic β-cells secrete insulin, which suppresses glucose production in the liver and stimulates glucose uptake in muscle and adipose tissue. In the fasted state, glucagon, epinephrine, and glucocorticoids increase hepatic glucose production directly or indirectly through the cAMP-PKA signaling pathway to maintain euglycemia (5).

FOXO proteins include FOXO1, 3, 4, and 6 and are members of the Fox superfamily (6). Mounting evidence suggests that FOXO proteins play a critical role in metabolism and energy homeostasis. For example, FOXO members up-regulate gluconeogenesis through the activation of *Pck1* and *G6pc* gene expression in the liver (7–9), activate pancreatic β-cell function (10), and promote differentiation of adipocytes (11). FOXO1 is particularly important in inhibition of hepatic gluconeogenesis by insulin; insulin inhibits FOXO1 activity through the PI3K/AKT signaling pathway (12, 13). Another layer of FOXO1 regulation is via acetylation of the cAMP-response element-binding protein (CREB)^3^ co-activators P300 and CBP (14–17).

Phosphorylation of FOXO1 by insulin leads to its nuclear exclusion and degradation in the fed state (12, 13), yet the mechanism driving *Foxo1* expression in the fasted state remains unclear. Glucagon activates the cAMP-PKA signaling pathway, and phosphorylation of CREB at Ser-133 by PKA, in turn, recruits the CREB co-activators CBP, P300, and CRTC2 to CRE-containing genes and activates hepatic gluconeogenesis (18, 19). However, we have reported previously that CBP phosphorylation at Ser-436 by insulin in the fed state triggers the disassembly of the CREB-CBP-CRTC2 complex (18) and inhibits hepatic glucose production. Furthermore, phosphorylation of CRTC2 at Ser-171 by insulin leads to its nuclear exclusion and degradation (19). Considering these studies, we wanted to test the hypothesis that elevated fasting glucagon levels increase *Foxo1* gene expression through recruitment of CREB co-activators. In this study, we have examined the potential role of CREB co-activators in increasing *Foxo1* gene expression in *in vitro* and *in vivo* experiments that model the fasting state.

## EXPERIMENTAL PROCEDURES

### 

#### 

##### Plasmids and Adenoviruses

The expression vectors for P300 and PKA used here were described previously (20). Mous*e Foxo1* gene promoter-luciferase reporters were constructed by cloning the promoter of *Foxo1* (up to −2000 to +1) into the pGL4 luciferase reporter construct. The BLOCK-iT adenoviral RNAi expression system (Invitrogen) was used to construct adenoviral shRNA for CBP, P300, CREB, and scrambled shRNA as we described previously (18).

##### Cell Cultures

Equal amounts of plasmids were transfected using Lipofectamine 2000 (Invitrogen) or adenoviral shRNAs into mouse hepatoma Hepa1–6 cells. After 48 h of incubation, cells were exposed to 0.2 mm dibutyrl cAMP for 5 h, 20 μm P300-specific histone acetyltransferase (HAT) inhibitor C646 or its inactive C37 analog (21). The C37 analog differs from C646 by only one double bond but is completely silent as a P300 inhibitor, serving to control for off-target effects of C646 (22).

##### Glucose Production Assays

Mouse primary hepatocytes were cultured in 6-well plates with William's medium E supplemented with ITS (BD Biosciences) and 27.5 nm dexamethasone. 18 h after the planting, primary hepatocytes were treated with 20 μm C37 or C646 for 3 h during serum starvation. Then, medium was replaced with 1 ml of glucose production buffer consisting of glucose-free DMEM supplemented with 20 mm sodium lactate and 2 mm sodium pyruvate or with 0.2 mm 8-bromo-cAMP and 20 μm C37 or C646 chemicals, and incubated for another 3 h.

##### Animal Experiments

All animal protocols were approved by the Institutional Animal Care and Use Committee of the Johns Hopkins University. C57BL/6 mice were purchased from The Jackson Laboratory, and 10-week-old mice were used. Mice were given C37 or C646 (30 nmol/g) through intraperitoneal injection and then subjected to fasting. Mice were sacrificed after an 8-h fast. In adenoviral shRNA knockdown experiments, 48 h after mice were injected with the adenovirus through tail vein, mice were subjected to an 16-h fast before being sacrificed (23).

##### Immunoblotting, Real-time qPCR, and Chromatin Immunoprecipitation

Immunoblotting was conducted as described previously (18, 20). Cellular lysates were sonicated for 15 s three times sequentially and immunoblotted to examine the target proteins with antibodies against CBP, P300 (Santa Cruz Biotechnology), CREB, pCREB, and FOXO1 (Cell Signaling) at concentrations recommended by the manufacturers. Secondary antibodies were used at the concentrations of approximately 1:5000. The primers used for the measurement of mouse *Foxo1* mRNA in real-time qPCR were as follows: 5′-primer (5′-ACATTTCGTCCTCGAACCAGCTCA-3′) and 3′-primer (5′-ATTTCAGACAGACTGGGCAGCGTA-3′). The chromatin immunoprecipitation assay was performed as we described previously (20); eluted DNA was amplified by real-time PCR with a 5′-primer (5′-TACCCCACCGCCCCCCACCAA-3′) and a 3′-primer (5′-GACTGACAGGCTGCGCGGCCA-3′) specific for the CRE-containing regions in the mouse *Foxo1* promoter. To examine the specific binding in ChIP assay, eluted DNA was also amplified with primers targeting *Gapdh* promoter, 5′-primer (5′-ACCTCAACTACATGGTCTACATGTT-3′) and 3′-primer (5′-CAAACATGGGGGCATCGGCAGAA-3′).

##### Statistical Analyses

Statistical significance was calculated with Student's *t* test and analysis of variance. Significance was accepted at the level of *p* < 0.05.

## RESULTS

### 

#### 

##### Fasting Resulted in a Marked Increase of Hepatic Foxo1 mRNA and FOXO1 Protein Levels

**FIGURE 1.**
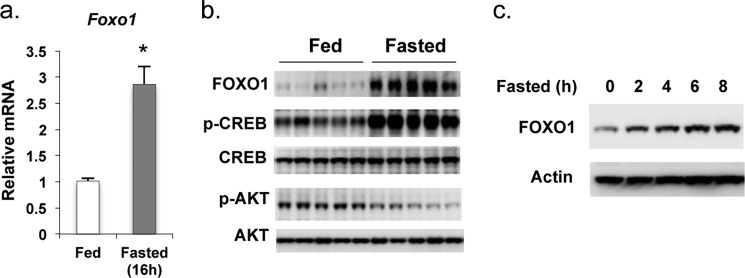
**Fasting induces *Foxo1* gene expression.** *a*, *Foxo1* mRNA levels compared in the liver of mice sacrificed at fed or fasted (16 h) states (*n* = 5). Real-time qPCR was used to measure gene expression (normalized to 36B4 expression levels). *Asterisk* (*) signifies that groups with same treatment are significantly different (*p* < 0.05). *Error bars*, S.E. *b*, phosphorylation status of CREB, AKT and total AKT, CREB, and Foxo1 protein levels in the liver from fed and 16-h fasted mice are shown (*n* = 5). *c*, fasting led to the early induction of Foxo1 expression. The Foxo1 protein levels in the liver of mice sacrificed are shown at the indicated fasting time points. Each *lane* represents sample pooled from two mice.

Because phosphorylation of FOXO1 by activated AKT in the insulin signaling pathway results in FOXO1 nuclear exclusion and degradation in the fed state (12, 13), it is conceivable that fasting might increase FOXO1 levels due to its important role in stimulating gluconeogenesis and maintaining euglycemia (7–9). Indeed, hepatic *Foxo1* mRNA levels increase approximately 3-fold in the liver of mice after 16 h of fasting (Fig. 1*a*). FOXO1 protein levels were also markedly increased in the fasted state, which was associated with an increase in phospho-CREB and a decrease in phospho-AKT (Fig. 1*b*). In a fasting time course experiment, FOXO1 protein levels increased as early as 2 h after the start of fasting and steadily increased in the fasting period (Fig. 1*c*).

##### Dibutyrl cAMP (Bt-cAMP) Stimulated the Foxo1 Gene Expression in Hepatocytes

**FIGURE 2.**
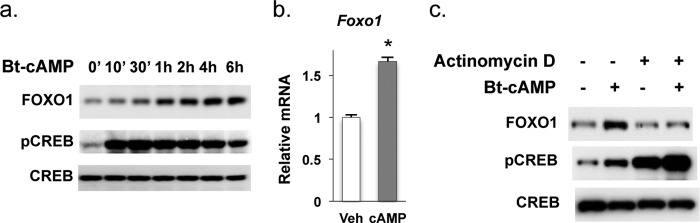
**Activation of cAMP-PKA pathway leads to the expression of Foxo1 gene.** *a* and *b*, 16 h after the seeding, Hepa1–6 cells were subjected to serum starvation in FBS-free DMEM for 3 h, then 1 mm Bt-cAMP was added and cells were incubated for 5h before harvesting. *b*, *Foxo1* mRNA levels were measured by using real-time qPCR, and data were normalized to 36B4 expression levels) (*n* = 3). *Asterisk* (*) signifies that groups with same treatment are significantly different (*p* < 0.05). *Error bars*, S.D. *c*, Hepa1–6 cells were treated with actinomycin D (2.5 μg/ml) for 30 min in FBS-free DMEM prior to the addition of 1 mm Bt-cAMP (6 h).

To test whether the activation of cAMP-PKA pathway is able to increase the expression of the *Foxo1* gene, we treated hepatoma Hepa1–6 cells with Bt-cAMP, a nonhydrolyzable cAMP analog that activates the cAMP-PKA signaling pathway. As shown in Fig. 2, Bt-cAMP treatment led to increased FOXO1 protein (Fig. 2*a*) as well as *Foxo1* mRNA levels (Fig. 2*b*). Furthermore, the inhibition of *Foxo1* transcription by actinomycin D blocked induction of FOXO1 protein levels by cAMP (Fig. 2*c*). These data suggest that the cAMP-PKA pathway activates *Foxo1* gene expression and that the activation of the gluconeogenic enzyme profile by glucagon stimulation during fasting is mediating at least in part by an increase in *Foxo1* gene expression.

##### CREB Co-activator P300 Regulates Foxo1 Gene Expression

**FIGURE 3.**
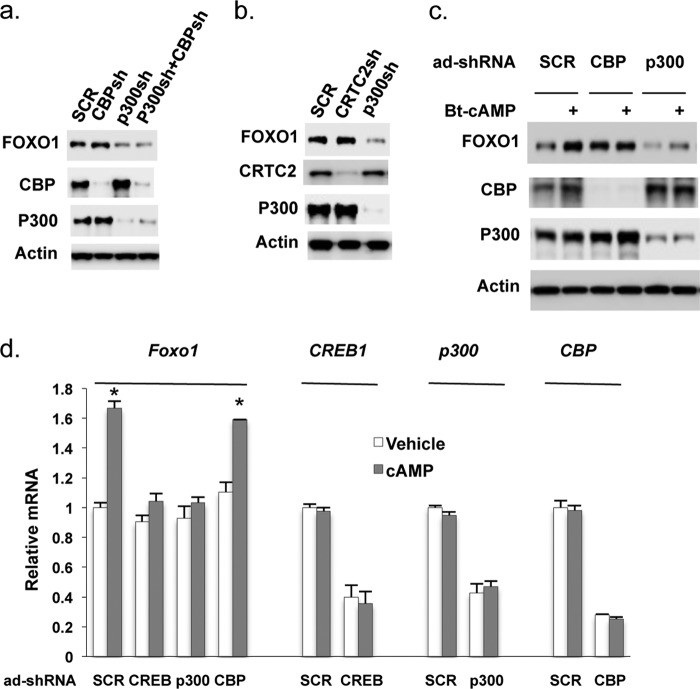
**Co-activator P300 mediates the *Foxo1* gene expression.** *a* and *b*, 48 h after the addition of adenoviral shRNAs to deplete CBP, P300, and CRTC2, Hepa1–6 cells were then treated with 0.2 mm Bt-cAMP for 6 h. Immunoblot analyses were conducted to determine the protein levels using indicated antibodies. *c*, 48 h after adenoviral shRNAs mediated depletion of CBP or P300, primary hepatocytes were subjected to serum starvation for 3 h. After washing with PBS, 0.2 mm Bt-cAMP was added in glucose production medium. *d*, in Hepa1–6 cells, 48 h after the addition of adenoviral shRNAs of scrambled, CREB, CBP, and P300, growth medium was changed to FBS-free DMEM and incubated for 3 h, then 1 mm Bt-cAMP was added and incubated for 5 h before harvesting. Real-time qPCR was used to measure gene expression (*n* = 3), and data were normalized to 36B4 expression levels. Each *lane* represents sample pooled from three treatments. *Asterisk* (*) signifies that groups with same treatment are significantly different (*p* < 0.05). *Error bars*, S.D.

Having seen that Bt-cAMP treatment increased *Foxo1* gene expression (Fig. 2), we investigated the potential involvement of the CREB co-activators CBP, P300, and CRTC2 in regulating *Foxo1* gene expression. We employed adenoviral shRNAs to deplete these co-activators individually in Hepa1–6 cells. Depletion of CBP and CRTC2 did not change the FOXO1 protein levels compared with cells treated with scrambled shRNA adenovirus (Fig. 3, *a* and *b*). In contrast, depletion of P300 decreased FOXO1 protein levels.

We examined further the role of CBP and P300 in the regulation of FOXO1 expression in primary hepatocytes. As shown in Fig. 3*c*, cAMP treatment increased FOXO1 protein levels in hepatocytes treated with scrambled shRNA adenovirus. Depletion of P300 by adenoviral shRNA blocked the induction of FOXO1 by Bt-cAMP. In comparison, depletion of CBP did not affect the overall protein levels of FOXO1. Moreover, depletion of P300 and CREB also blocked the induction of *Foxo1* mRNA by Bt-cAMP. Again, depletion of CBP did not significantly affect *Foxo1* mRNA levels in Hepa1–6 cells (Fig. 3*d*). The above data suggest that cAMP-induced *Foxo1* gene expression is mediated by CREB and P300.

**FIGURE 4.**
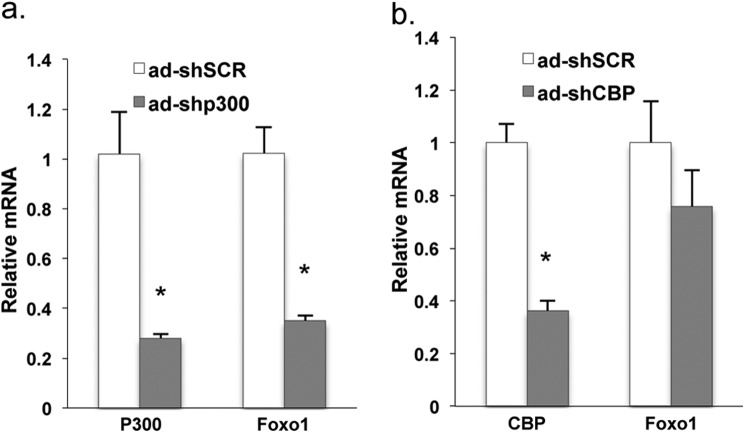
**Depletion of P300 decreased Foxo1 mRNA levels in the liver.** *Foxo1* mRNA levels in the liver of mice with adenoviral shRNAs mediated depletion of P300 (*a*) or CBP (*b*), and sacrificed after 16 h fasting (*n* = 3). *Asterisk* (*) signifies that groups with the same treatment are significantly different (*p* < 0.05). *Error bars*, S.D.

Given the importance of CBP and P300 in regulation of gluconeogenic gene expression in the liver, we next assessed the role of CBP and P300 in regulation of *Foxo1* gene expression *in vivo*. We used adenoviral shRNAs to deplete CBP or P300 in the liver of mice through tail vein injection. Depletion of P300 significantly decreased *Foxo1* mRNA levels in the liver of fasted mice (Fig. 4*a*), whereas the depletion of CBP had a minimal effect on *Foxo1* mRNA levels (Fig. 4*b*).

##### The Foxo1 Promoter Contains CREs

**FIGURE 5.**
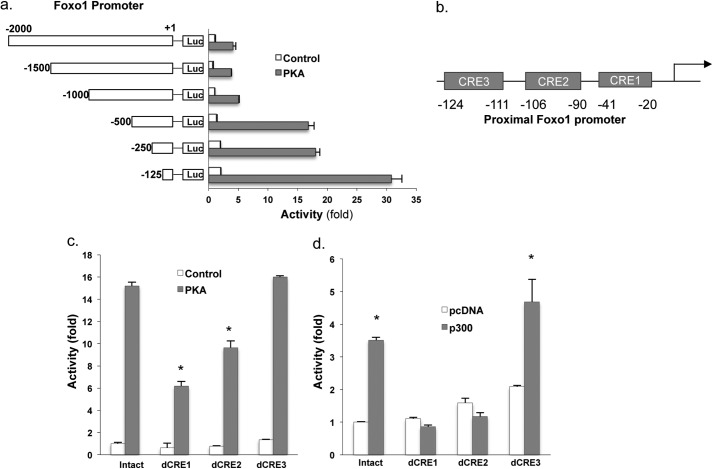
**Identification of the functional regions in *Foxo1* gene promoter.** *a*, schematic shows reporter constructs tested. Nucleotides are *numbered* relative to the transcription start site (bp +1). 20 ng of reporter constructs was transfected into Hepa1–6 cells together with 400 ng of control RSV-cat or PKA expression plasmids. Reporter activities were measured 48 h after the transfection. *b*, three CRE site were identified in the proximal promoter region of the *Foxo1* gene. *c*, three CREs were individually deleted in the context of the proximal promoter construct (−125 bp) and tested again. Hepa1–6 cells were transfected with 20 ng of each construct together with 200 ng of control RSV-cat or PKA expression plasmids. *d*, 20 ng of the proximal promoter construct and its mutants and 200 ng of PKA expression plasmid were co-transfected into Hepa1–6 cells together with 400 ng of control pcDNA or P300 expression vector. Reporter activities were measured 48 h after the transfection. *Asterisk* (*) signifies that groups with same treatment are significantly different (*p* < 0.05). *Error bars*, S.D.

To characterize the *Foxo1* promoter, a series of mouse *Foxo1* promoter luciferase constructs were made and tested in Hepa1–6 cells. Fig. 5*a* is an illustration of the construct used and the 5′-end point. Reporter activity is relative to the −2000-bp construct, which was assigned a value of 1 in the absence of co-transfected PKA (*white bars*). PKA co-transfection increased promoter activity of all constructs, suggesting that a CRE may be located in the proximal 125 bp of the promoter. After analysis of the −125 bp region of the *Foxo1* proximal promoter using the TransFac Database, three putative CREs were identified as shown and labeled CRE1, 2, and 3 (Fig. 5*b*). To define the role of these CREs on the *Foxo1* gene in cAMP-PKA induction, these CREs were individually deleted in the context of a −125-bp promoter construct and tested again in Hepa1–6 cells. Deletion of either CRE1 or CRE2 significantly reduced PKA-stimulated reporter activity (Fig. 5*c*), whereas deletion of CRE3 had no effect on the promoter activity. By using these constructs, we assessed further their effect on reporter activity after co-transfection with P300 and PKA expression plasmids in Hepa1–6 cells. In the wild type construct, P300 co-transfection markedly increased promoter activity, whereas deletion of either CRE1 or CRE2 in the reporter construct negated the P300 effect (Fig. 5*d*). Deletion of CRE3 had no effect. Because P300 is known to interact with CREB, these data suggest that P300 may interact with CREB and bind to CRE1 and CRE2 of the *Foxo1* promoter.

##### Recruitment of P300 to the Foxo1 Promoter after PKA Stimulation

**FIGURE 6.**
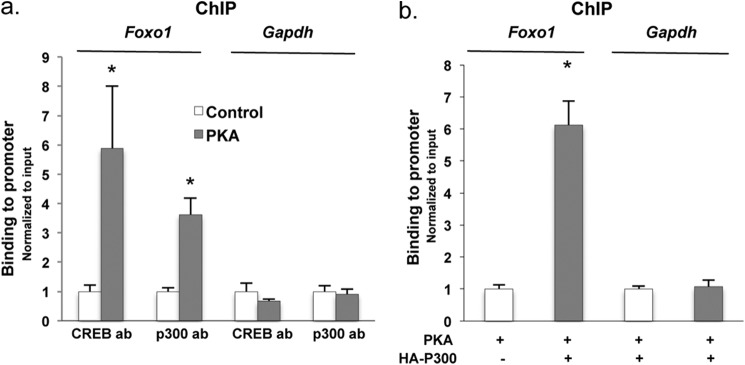
**P300 binds to the proximal promoter region of the *Foxo1* gene.** *a*, PKA stimulated the binding of CREB and p300 to CREs in the proximal promoter of the *Foxo1* gene. 48 h after the transfection of PKA expression plasmid, Hepa1–6 cells were fixed with formaldehyde followed by immunoprecipitation with CREB and P300 antibodies. The DNA in the immunoprecipitates was amplified using primers encompassing 150 bp including the CREs in the promoter of the mouse *Foxo1* gene (*n* = 3). *b*, Hepa1–6 cells were transfected with PKA or together with HA-tagged P300 expression plasmid, followed by the fixation and immunoprecipitation using anti-HA tag-specific antibody. The binding of HA-tagged P300 was determined by real-time PCR (*n* = 3). *Asterisk* (*) signifies that groups with same treatment are significantly different (*p* < 0.05). *Error bars*, S.D.

To investigate whether recruitment of CREB and P300 to CREs was induced by PKA stimulation, we conducted a ChIP assay. Hepa1–6 cells were transfected with control or PKA-containing plasmids to assess the occupancy of CREB and P300 on *Foxo1* and a negative control *Gapdh* promoter. Fig. 6*a* shows the quantitative PCR for *Foxo1* or the negative control *Gapdh* promoter of either CREB- or P300-immunopreciptated chromatin. PKA transfection markedly increased the occupancy of both CREB and P300 on the *Foxo1* promoter but not on the negative control *Gapdh* promoter. These data correlate with the functional data obtained in the same mouse hepatocyte cultures as shown in Fig. 5. To probe further whether PKA stimulated the occupancy of P300 on the proximal *Foxo1* promoter, we overexpressed HA-tagged P300 together with PKA and used a HA tag-specific antibody to immunoprecipitate the cross-linked *Foxo1* promoter in a ChIP assay. The binding of HA-tagged P300 increased >6-fold in the presence of PKA stimulation (Fig. 6*b*).

##### HAT Activity of P300 Is Important for Regulating Foxo1 Gene Expression

**FIGURE 7.**
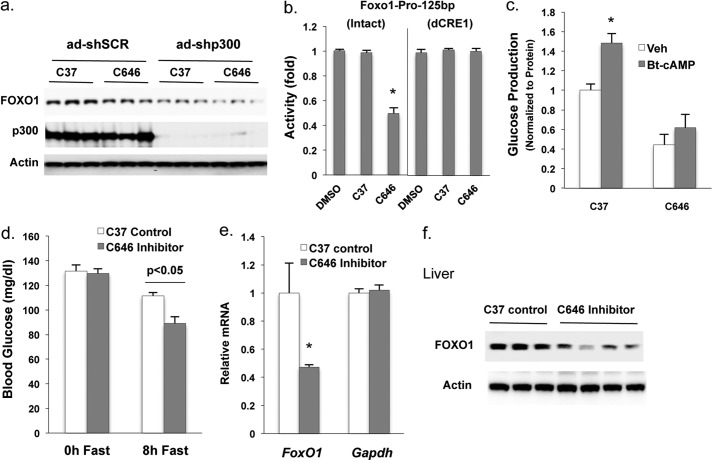
**P300 binds to the proximal promoter region of the *Foxo1* gene.** *a*, 48 h after the addition of adenoviral shRNAs, Hepa1–6 cells were grown in FBS-free DMEM supplemented with 10 μm control C37 or HAT inhibitor C646 for 3 h, followed by the addition of 0.2 mm Bt-cAMP and incubated for 6 h. *b*, Hepa1–6 cells were transfected with 20 ng of promoter constructs, 400 ng of PKA, and 500 ng of P300 expression plasmids. 24 h later, 20 μm C37 or C646 was added, and cells were incubated for another 24 h. *c*, in primary hepatocytes, 20 μm C37 or C646 was added to FBS-free medium during serum starvation. After washing with PBS, 0.2 mm Bt-cAMP together with 20 μm C37 or C646 was added in glucose production medium for another 3 h. *d–f*, administration of HAT inhibitor significantly decreased fasting blood glucose levels (*d*), and hepatic *Foxo1* mRNA levels (*e*), as well as the protein levels (*f*) of mice (*n* = 3∼4). *Asterisk* (*) signifies that groups with same treatment are significantly different (*p* < 0.05). *Error bars*, S.D.

P300 contains intrinsic HAT activity, and histone acetylation by this co-activator leads to the chromatin remodeling and an increase in gene transcription (24). To test whether P300 HAT activity has a role in regulating *Foxo1* gene expression, adenoviral shRNA was employed to deplete P300 in Hepa1–6 cells. Then, cells were treated with Bt-cAMP and/or the HAT inhibitor C646, which is a P300 HAT-specific inhibitor (21, 22). C646 treatment decreased FOXO1 protein levels, demonstrating that the HAT activity of P300 is indeed important in *Foxo1* gene expression (Fig. 7*a*). Moreover, P300 is also important in regulating basal expression of the *Foxo1* gene as shown in the shRNA knockdown of P300. We next sought to determine the mechanism of HAT inhibition on *Foxo1* gene expression. Using *Foxo1* luciferase reporters, we show that the HAT inhibitor C646 significantly decreased PKA-stimulated reporter activity, whereas a CRE mutant construct was not affected (Fig. 7*b*). A control compound C37 without HAT inhibitory properties was without effect. Moreover, treatment with C646 markedly decreased both basal and Bt-cAMP-stimulated glucose production in primary hepatocyte, whereas the Bt-cAMP-stimulated glucose production was intact after treatment with the control compound C37 (Fig. 7*c*). Finally, mice administrated with C646 through intraperitoneal injection exhibited significantly lower fasting blood glucose levels (Fig. 7*d*) accompanied by decreased levels of *Foxo1* mRNA and FOXO1 protein levels (Fig. 7, *e* and *f*).

## DISCUSSION

Blood glucose levels are finely regulated by the opposing actions of the insulin and glucagon signaling pathways. In the fed and postprandial states, the uptake of glucose from the gastrointestinal tract causes elevation in blood glucose levels, which in turn, triggers the secretion of insulin from pancreatic β-cells. Insulin stimulates glucose utilization in the muscle and adipose tissue and suppresses endogenous glucose production in the liver, so that glucose eventually returns to baseline levels. The molecular mechanism for the suppression of hepatic glucose production by insulin is complicated and likely involves a number of transcriptional factors and co-activators, and several mechanisms have been reported. One mechanism involves phosphorylation of FOXO1 by insulin through PI3K-AKT, which excludes FOXO1 from nucleus and promotes cytoplasmic ubiquitinylation and degradation (25, 26). Another mechanism suggests that CRTC2 may undergo a similar exclusion from the nucleus after phosphorylation of Ser-171 by the insulin (19). Our laboratory has proposed a third mechanism to ensure a sufficient control of hepatic gluconeogenesis, where insulin regulates the composition of CREB co-activator complex. Phosphorylation of CBP at Ser-436 by insulin leads to the disassembly of the CREB-CBP-CRTC2 complex (18).

**FIGURE 8.**
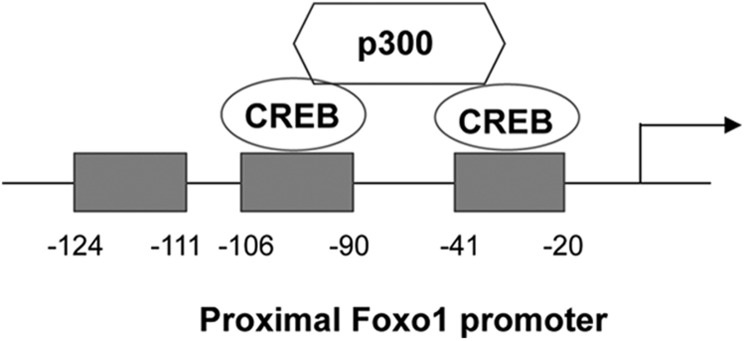
**Proposed model for the regulation of *Foxo1* gene by P300.**

In the fasting state, glucagon activates phosphorylation of CREB at Ser-133 through a cAMP-PKA pathway, which leads to recruitment of the co-activators CBP, P300, and CRTC2 to CRE-containing genes such as *Pck1* and *G6pc*. The increased expression of gluconeogenic genes is thought to be a major way in which glucagon increases hepatic gluconeogenesis (18, 19); and accordingly, FOXO1 induces *Pck1* and *G6pc* gene expression (8, 9). In light of this information, we reasoned that glucagon might increase *Foxo1* gene expression in the fasted state through CREB co-activators. In this way, FOXO1 would further enhance gluconeogenesis. We found that depletion of CREB and P300 abolished the induction of *Foxo1* mRNA by cAMP (Fig. 3*d*); however, depletion of CBP and CRTC2 had no effect on *Foxo1* mRNA and FOXO1 protein induction by cAMP (Fig. 3, *a–d*). These data suggest that CREB-P300 mediates the *Foxo1* induction, revealing a unique role for P300 in regulating *Foxo1* gene expression (Fig. 8). This notion was further substantiated by data obtained after depletion of hepatic CBP and P300 in the fasted mice, in which only the depletion of p300 resulted in a significant decrease in *Foxo1* mRNA (Fig. 4). P300 has HAT activity, and in accordance with a previous report, the inhibition of HAT activity of CBP and/or P300 significantly decreased fasting blood glucose levels (Fig. 7*c*) (8). These effects might be mediated, in part, through the suppression of *Foxo1* gene expression, because P300 is known to regulate *Foxo1* gene expression (14–16). Indeed, administration of a P300 HAT-specific inhibitor C646 decreased reporter activity (Fig. 7*b*) and lowered the fasting blood glucose levels together with decreasing *Foxo1* mRNA and FOXO1 protein levels (Fig. 7, *e* and *f*). Although we report here that the HAT activity of P300 is important for the transcription of *Foxo1* gene, the specific acetylation target may involve lysines on histones or other proteins. For example, the HAT activity of P300 might acetylate FOXO1 protein and affect its protein stability because deacetylation of FOXO by sirtuin 1 reduces levels of mammalian FOXO transcriptional factors (27–29). Of note, some investigators suggest that the acetylation of FOXO1 facilitates FOXO1 phosphorylation by insulin, which should reduce FOXO1 levels by promoting its nuclear exclusion (17, 30).

In summary, we demonstrate that the P300 specifically activates *Foxo1* gene expression at the transcriptional level. Previous reports have shown that insulin signaling terminates nuclear FOXO1 action by phosphorylating the protein, which promotes its nuclear exclusion and degradation. This is the first report to our knowledge, however, to show that co-activator P300 mediates a large increase in FOXO1 protein levels during fasting state and suggests that FOXO1 might have a much greater role in regulating gluconeogenesis in the fasting state.

## Footnotes

3 The abbreviations used are:

CREB: cAMP-response element-binding protein
Bt-cAMP: dibutyrl cAMP
CBP: CREB-binding protein
CRE: cAMP-response element
HAT: histone acetyltransferase
qPCR: quantitative PCR.
